# The application of sigma metrics in the laboratory to assess quality control processes in South Africa

**DOI:** 10.4102/ajlm.v11i1.1344

**Published:** 2022-06-22

**Authors:** Marli van Heerden, Jaya A. George, Siyabonga Khoza

**Affiliations:** 1 National Health Laboratory Service, Johannesburg, South Africa; 2 Faculty of Health Sciences, Charlotte Maxeke Johannesburg Academic Hospital, University of the Witwatersrand, Johannesburg, South Africa

**Keywords:** quality control, diagnostic errors, laboratories, clinical chemistry tests, sigma metrics, Six Sigma, chemical pathology

## Abstract

**Background:**

Laboratories use quality control processes to monitor and evaluate analytical performance in terms of precision and bias. Sigma metrics provide an objective assessment of laboratory quality using the total allowable error as an additional parameter.

**Objective:**

This study aimed to determine the sigma metrics of analytes when using different total allowable error guidelines.

**Methods:**

A retrospective analysis was performed on 19 general chemistry analytes at Charlotte Maxeke Johannesburg Academic Hospital in South Africa between January 2017 and December 2017. Sigma metrics were calculated on two identical analysers, using internal quality control data and total allowable error guidelines from the Ricos biological variation database and three alternative sources (the Royal College of Pathologists of Australasia, the Clinical Laboratory Improvements Amendment, and the European Federation of Clinical Chemistry and Laboratory Medicine).

**Results:**

The sigma performance was similar on both analysers but varied based on the guideline used, with the Clinical Laboratory Improvements Amendment guidelines resulting in the best sigma metrics (53% of analytes on one analyser and 46% on the other had acceptable sigma metrics) and the Royal College of Pathologists of Australia guidelines being the most stringent (21% and 23%). Sodium and chloride performed poorly across all guidelines (sigma < 3). There were also month-to-month variations that may result in acceptable sigma despite poor performance during certain months.

**Conclusion:**

The sigma varies greatly depending on the total allowable error, but could be a valuable tool to save time and decrease costs in high-volume laboratories. Sigma metrics calculations need to be standardised.

## Introduction

Medical laboratories strive to produce accurate reproducible results as physicians rely on these for diagnosis, monitoring, and prognostication of patients.^1^ To produce results with no errors, medical laboratories thus monitor and evaluate analytical processes using several different quality control (QC) processes. In practice, there are no processes with zero defects.^2^

The performance of analytical procedures is typically evaluated in terms of precision and accuracy (bias). This is determined using QC procedures performed at intervals as determined by laboratory policy. A high standard deviation indicates poor precision, instability, and high random error.^3^ Most South African laboratories use Levey-Jennings control charts and Westgard QC rules to determine whether a QC run is acceptable based on an algorithm with specified limits. This approach might not be ideal, as one set of rules cannot be applied to all tests due to varying precision and goals.^4^

The number of QC levels and the frequency of QC runs varies greatly between laboratories.^5^ The National Accreditation Board for Testing and Calibration Laboratories guidelines report that two-level controls should be run at a peak hour and, subsequently, one level every 8 h for laboratories that run continuously.^6^ Different rules may be applied to determine if the QC values are acceptable or not. Most laboratories use 1_2_S as a warning rule. This implies that a single control measurement exceeding two standard deviations from the mean (in any direction) may indicate a problem.^5^ However, when this rule is used as a control rule, it can cause a false rejection rate of up to 14%.^5^ Internal QC policies regard the 1_3_S, R_4_S and 2_2_S rules as criteria for rejection, while ten consecutive observations on one side of the mean (10 × rule) require further investigation. Combinations of rules (multirules) are sometimes employed to reduce the rate of false rejections and to save time and effort by incorporating rules that are sensitive to both random and systematic errors.

Six Sigma further elaborates on this by individualising control rules based on the analytical performance of the test.^7^ Six Sigma is a QC strategy where a statistical calculation is performed to evaluate the effectiveness of laboratory processes. The sigma scale provides an objective manner to assess and compare laboratory quality by incorporating both the imprecision and bias observed in a laboratory’s performance.^8^ The sigma metric is based on three parameters: total allowable error (TEa), bias and imprecision. The TEa guidelines from various sources are associated with significantly different sigma metrics for the same assay.^9,10^

Six Sigma can be used to decide on the best Westgard rule by judging the performance of a process against a reference method and assessing the quality of laboratory processes, thereby identifying processes needing improvement. As demonstrated by Litten,^7^ the implementation of a Six Sigma-designed QC programme can result in fewer controls per run, fewer false rejections, simpler Westgard rules, and a 45% saving on laboratory reagents and supplies.

Our laboratory runs two identical analysers in parallel and currently does not use sigma metrics to manage QC. There has been a big shift in focus towards quality laboratory improvement, especially in developing countries. There are limited studies on sigma metric performance in South African laboratories. This study thus aimed to determine the variations in sigma metrics of selected analytes with different TEa guidelines and to assess if sigma differed between two identical analysers.

## Methods

### Ethical considerations

Ethical approval, in the form of a waiver, was obtained from the University of the Witwatersrand Human Research Ethics Committee (number: W-CBP-180216-01). The results of quality control samples were utilised and therefore no patient consent was required.

### Study setting

This retrospective study was conducted in a National Health Laboratory Service laboratory at a large academic tertiary hospital in South Africa, which is accredited to International Organization for Standardization standard 15189. There are two identical Cobas^®^ 8000 chemistry analyser (Roche Diagnostics, Mannheim, Germany) systems (referred to as Analyser 1 and Analyser 2) running in parallel in the laboratory, with some tests run on both analysers.

### Study design

A retrospective analysis of 19 analytes was performed using internal QC data obtained from the Roche Cobas^®^ 8000 chemistry analyser IT middleware system (Roche Diagnostics, Mannheim, Germany) over 12 months (January 2017 – December 2017). These analytes were chosen as they are routinely analysed and can be compared to studies in the literature. The laboratory used Roche QC materials to perform the internal QC testing. Two levels of internal QC are performed for each analyte, one in the normal range and one in the abnormal (high) range. The QC materials used include Roche Cobas^®^ PreciControl ClinChem Multi 1 and 2, PreciControl Tumor Marker, and PreciControl Universal. A single lot of QC materials, except for prostate-specific antigen (PSA) and thyroid-stimulating hormone (TSH), was used throughout the 12 months.

The following tests were included in our study: alanine aminotransferase, alkaline phosphatase, aspartate aminotransferase (AST), calcium, chloride, cholesterol, creatine kinase (CK), creatinine, direct bilirubin (DBIL), glucose, high-density lipoprotein cholesterol, potassium, sodium, TSH, total bilirubin, total protein, PSA, urea, and uric acid. The reagents, QC materials and calibrator materials were all provided by Roche Diagnostics, Mannheim, Germany.

### Data collection and analysis

Using internal QC means and standard deviations, we calculated the bias and coefficient of variation (CV). The bias was determined by subtracting the packing insert target value (mean) from the observed QC mean. The CV was determined using the following formula:

CV=100×(standard deviation/mean). [Eqn 1]

Outliers (values exceeding 1_3_S) were excluded using the Cobas IT middleware.

For our study, the biological variation (BV) database from Ricos and colleagues, which was last updated by the Spanish Society of Laboratory Medicine in 2014,^11^ was used to determine the desirable test-specific quality requirements. This database was compared to the TEa guidelines from the Clinical Laboratory Improvement Amendments (CLIA),^12^ the Royal College of Pathologists of Australasia (RCPA),^13^ and the European Federation of Clinical Chemistry and Laboratory Medicine (EFLM).^14^

TEa values given as percentages were converted to units with the following calculation:

TEa (units)=(TEa%/100%)*target value. [Eqn 2]

Sigma metrics were calculated for each analyte on two levels as follows:

Sigma=TEa−(bias/standard deviation). [Eqn 3]

Thereafter, the average annual sigma metric was obtained. The Quality Goal Index (QGI) indicates the possible source of error and represents the relative degree to which bias and precision meet their quality goals.^15^ The QGI was determined as follows:

QGI=Bias/1.5CV. [Eqn 4]

A QGI score of < 0.8 indicates imprecision, QGI scores between 0.8 and 1.2 indicate both imprecision and inaccuracy, and a score of > 1.2 indicates inaccuracy.^15^ Meta-analysis data for within- (CV_i_) and between- (CV_g_) subject variation were obtained from the EFLM Biological Variation database.^14^

The desirable specifications for imprecision, bias, and TEa were calculated as follows:

$$\begin{array}{l} \text{CV}\%=0.5({\text{CV}}_{\text{i}})\\ \text{Bias}\%=0.25({\text{CV}}_{\text{i}}^{2}+{\text{CV}}_{\text{g}}^{2}{)}^{0.5}\\ \text{TEa}\%=(1.65\times \text{CV}\%)+\text{Bias}\% \end{array}$$ [Eqn 5]

The allowable limits of performance for total bilirubin, DBIL, calcium, and uric acid were obtained from the Westgard website based on EFLM data.^16^

The normalised method decision charts were created by calculating and then plotting the observed inaccuracy (Bias% / TEa%) and the observed imprecision (CV% / TEa%).

Data capture and statistical analyses were performed using Windows^®^ 10, Microsoft Excel (Microsoft Corporation, Redmond, Washington, United States). The analyses performed include calculation of bias, CV, TEa, sigma metrics, and the QGI, as specified above.

## Results

**FIGURE 1:**
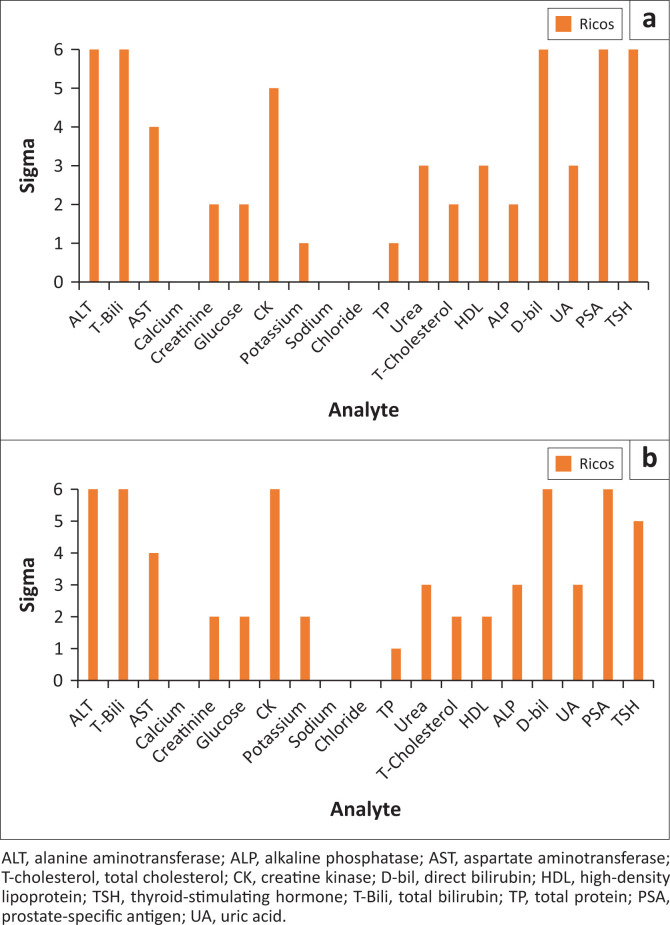
Average annual sigma of analytes tested on two chemistry analysers, Johannesburg, South Africa, January 2017 – December 2017. (a) Analyser 1; (b) Analyser 2.

Nine analytes (alanine aminotransferase, AST, total bilirubin, CK, Urea, DBIL, uric acid, PSA, and TSH) achieved sigma ≥ 3 on both analysers, while one analyte (uric acid) had a sigma of ≥ 3 on Analyser 1 and < 3 on Analyser 2 (Figure 1).

Performance was very similar across both analysers for each guideline. Using the Ricos database, ten analytes had a sigma of < 3 across both analysers, and four analytes had a sigma of > 6 on both analysers. Three analytes achieved sigma > 6 on both control levels, namely DBIL, PSA, and TSH on Analyser 1, as well as CK, DBIL, and PSA on Analyser 2 using Ricos (Online Supplementary Document Table 1). The sigma values for each analyte have been grouped according to performance for both analysers based on different sources of TEa and concentration of control material. The poorly performing analytes (with sigma values < 3) all had QGI values < 0.8, indicating imprecision as a possible cause.

Several analytes had sigma < 3 for both QC levels across both analysers. These included sodium, chloride (Online Supplementary Document Figure 1), glucose, and cholesterol. Only AST achieved a sigma of ≥ 3 with all guidelines and at both analyte concentrations, but this was seen only on Analyser 1. Aspartate aminotransferase and urea achieved sigma metrics between 3 and 6 on both analysers. No analyte achieved a sigma of > 6 across all guidelines and for two control levels. There were also large variations in performance from month-to-month. Many analytes achieved an acceptable annual average sigma despite poor or marginal performance during certain months. Examples include TSH, AST, total bilirubin and alanine aminotransferase. Conversely, CREA, CK level 1 and cholesterol level 2 failed to achieve acceptable sigma values, despite having sigma values ≥ 3 during certain months. Alanine aminotransferase, total bilirubin and AST level 1 on Analyser 1 (module 702) displayed similar patterns, with improvements noted during April, May, July, September, and November 2017.

**FIGURE 2:**
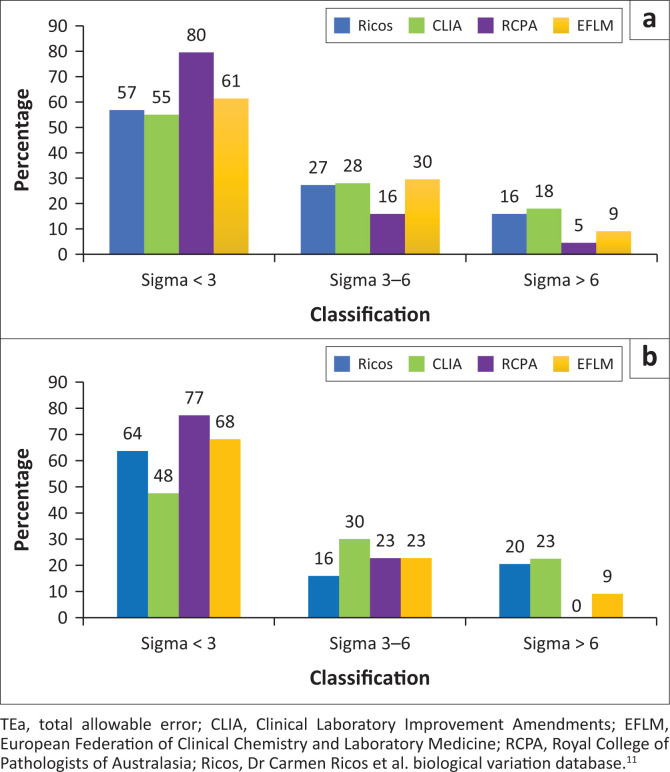
Sigma performance of analytes tested on two chemistry analysers based on different total allowable error guidelines, Johannesburg, South Africa, January 2017 – December 2017. (a) Sigma % per TEa guideline – Analyser 1; (b) Sigma % per TEa guideline – Analyser 2.

When comparing the sigma performance based on the different TEa guidelines, the CLIA BV guidelines resulted in the best sigma metrics, with 46% (Analyser 1) and 53% (Analyser 2) of analytes achieving sigma values ≥ 3 (Figure 2). Using the Ricos BV database, 43% of analytes on Analyser 1 and 36% on Analyser 2 had sigma values ≥ 3. Sixteen percent of analytes on Analyser 1 and 20% of analytes on Analyser 2 had sigma values > 6. With the EFLM guidelines, 39% of analytes on Analyser 1 and 32% on Analyser 2 had sigma values ≥ 3. The worst sigma metrics were obtained using RCPA guidelines: 21% of analytes had sigma ≥ 3 on Analyser 1 and 23% on Analyser 2. No analyte on Analyser 2 had sigma values > 6 using the RCPA guidelines.

**FIGURE 3:**
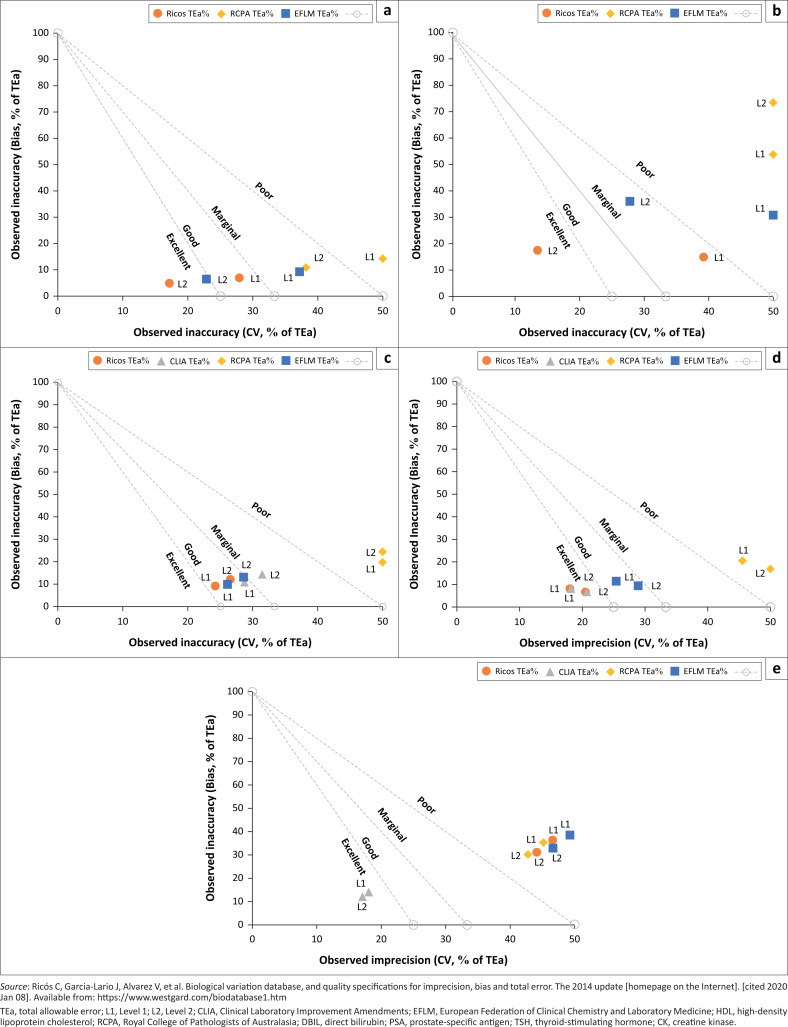
Normalised method decision chart for DBIL, PSA, TSH, CK, and HDL based on different total allowable error guidelines (Ricos, CLIA, RCPA, EFLM), Johannesburg, South Africa, January 2017 – December 2017. (a) DBIL Normalised Method Decision (Analyser 1); (b) PSA Normalised Method Decision (Analyser 1); (c) TSH Normalised Method Decision (Analyser 1); (d) CK Normalised Method Decision (Analyser 2); (e) HDL Normalised Method Decision (Analyser 2).

Based on the method decision charts for specific analytes on Analyser 1, more analytes were classified as excellent performers using the Ricos guidelines compared to RCPA (Online Supplementary Document Figure 2). The same pattern was noted for TSH on this analyser, with the EFLM guidelines also resulting in good sigma performance (Figure 3). There were also variations in performance between QC levels, with performance generally better in level 2. The Ricos and CLIA guidelines appear to be the most lenient, resulting in better performance for CK on Analyser 2. In general, the EFLM guidelines resulted in good sigma performance, while the RCPA guidelines, which are stricter, resulted in poor performance. High-density lipoprotein cholesterol generally performed poorly on Analyser 2, except when the CLIA guidelines were applied.

## Discussion

The analytical performance of a laboratory, as assessed by sigma metrics, has practical implications such as the design of QC programmes. In this study, QC data for 19 chemistry analytes collected over 12 months were analysed, and sigma was calculated for each analyte to objectively evaluate analytical performance. This study demonstrated that Six Sigma values vary depending on the TEa guidelines used, with analyte concentration, and from month-to-month.

There are no universally accepted TEa guidelines, and published data show that sigma metrics vary with the TEa guidelines used.^8,10,17^ There are several ways to address this. For example, Sharkawy et al.^17^ created a harmonisation protocol for sigma calculation to allow the comparison of sigma metrics across laboratories by using similar TEa guidelines. In this study, there were fewer differences across the two analysers using the same TEa guidelines compared to the sigma variations observed with different TEa guidelines. Another proposed approach is to assess the effect of TEa on patient outcomes. Researchers in China^18^ assessed the ‘severity of harm’ caused by TEa being exceeded in 36 analytes. A risk priority number was assigned by multiplying the sigma metric by the score of the intended use. The authors suggested that TEa should be defined by the highest possible hierarchical model and recommended that tests with negligible risks to patients be allowed to reach lower sigma metrics.^18^ In our study, the CLIA BV guidelines resulted in the best sigma metrics. This may change in the future as CLIA has proposed new limits that appear to be less lenient.^19^ The RCPA guidelines appeared to be the most stringent, which is in keeping with the results of a 2018 study performed in China.^20^ Recently, the EFLM has established the Working Group on Biological Variation and the Task and Finish Group for the Biological Variation Database to assess the quality of existing BV data and to compile global estimates in an attempt to harmonise analytical performance specifications worldwide.^21^ As proposed by Varela and Pacheco,^22^ another option would be to use an algorithm that standardises the procedure for the selection of the most appropriate TEa for evaluating analytical performance.

In addition to TEa, the analyte concentration is crucial when determining sigma metrics.^8^ In a study that investigated the performance of verified versus non-verified reagents, Cao et al.^23^ observed that sigma varied with analyte concentration and suggested that different rules be used for different analyte concentrations. The observed changes in sigma value with analyte concentration may be due to changes in precision and/or bias.^8^

There are different approaches to the calculation of bias and CV, which may both influence the final sigma calculation. For example, bias may be determined from external quality assessment reports rather than from internal QC data as done in this study. Guo et al.^9^ showed that both methods can be used for the determination of sigma metrics and suggest that laboratories evaluate sigma metrics multiple times to optimise QC schedules. Alternatively, bias may also be obtained from package inserts or can be derived from a group mean. The month-to-month variation noted with sigma was due to changes in CV and bias over these months. The CV, which is a measure of imprecision, was based on results over a 12-month period and therefore would be influenced by changes in reagents, calibrators, or personnel. It is therefore expected to be wider than CV determined over shorter periods and expected to have consequently lower sigma metrics.^24^ Current methods for the determination of sigma metrics have been criticised. According to Oosterhuys,^25^ the inclusion of bias in the calculation will result in an underestimation of analyte performance due to the short-term bias already reflected in the analytical imprecision. Westgard found that the assumption that bias should be ‘corrected or eliminated’ was invalid for many analytes as bias still exists despite attempts at standardisation.^25,26^

To address the variability of sigma performance between different QC levels, months and analysers, the average sigma or lowest sigma metric could be used for determining QC procedures.^27^ Westgard multirules^28^ will be easy to implement for well-performing analytes such as PSA and DBIL. It will however be complicated for analytes with low sigma metrics such as the electrolytes, as well as those performing differently on the different QC levels. The goal is to achieve 90% error detection and a 5% false rejection rate while using the lowest possible number of control rules.^7^ Operating specifications charts can be used to determine the number of control rules and the number of controls needed to achieve this goal.^29^ The lower the error detection, the more likely it is that more than one QC run will be required to detect a critical shift in performance and that erroneous patient results may be reported before the problem is detected.^30^ Moving average QCs have been suggested for high-volume analytes with low sigma metrics to decrease the risk of reporting erroneous results between scheduled QC runs.^31^

Our study demonstrated the need to increase internal QC and calibration frequency for some poorly performing analytes such as the electrolytes (sodium, potassium and chloride) regardless of the BV source used. The poor sigma performance of electrolytes is however not unique to our laboratory. Potassium, sodium, and chloride have low BV, and tight quality specifications are expected to give low sigma results.^27,32^ We used the QGI to investigate the reasons for low sigma performance (sigma values < 3) and showed that the main problem was imprecision. A search for new and improved calibration methods may improve the precision and, subsequently, the sigma metrics.^33^ When imprecision is poor relative to analytical goals, good error detection is hard to attain, regardless of the QC rules used.^30^

Ideally, sigma metrics should assist in decreasing operating costs by decreasing the amount of QC materials and reagents used and reducing unnecessary recalibrations.^29,34^ In addition, staff morale can improve by decreasing time spent troubleshooting and investigating false rejections.^29^ Zhou et al.^35^ compared new QC procedures (based on recommended error detection and false rejection criteria) with previous procedures adopted in their laboratory and found that for analytes with sigma values > 6, there is cost reduction and increased efficiency.

As poor-performing analytes will require the maximum number of control rules and control measurements per run, this can prove to be too expensive in our setting. Westgard found that for analytes with sigma values < 3, a full multirule procedure with at least four control measurements per run is required.^29,36^ Some authors find these stricter QC procedures to be unpractical because of the significant increase in the number of runs, especially when multiple analytes demonstrate sigma values < 4. However, when the quality of clinical results and benefits to patients are considered, the associated costs can be justified if reasonable.^35^

One of the strengths of this study is the 12-month duration; this gave a good reflection of the data as estimations of accuracy and imprecision are expected to improve with more data points.^30^ We also looked at the sigma performance of multiple analytes on two levels of QC to assess different modules on the instrument and to determine if the control level is a contributing factor. As the same lot of QC materials was used in this study, performance-related changes could be attributed to other factors. Comparing two identical analysers allowed us to assess the performance of analysers operating under the same environmental conditions and improved the consistency of our findings.

Laboratories should explore the practicality and feasibility of introducing sigma metrics as part of routine QC practice and for the review of poorly performing methods. We also recommend the careful selection of TEa guidelines and the standardisation of sigma metric calculations in the future.

### Limitations

One limitation of this study is that no third-party QC materials were used. The recommendation is that, when possible, third-party QC materials should be used rather than control materials supplied by the manufacturer.^30^ When QC materials are different from calibrator materials, it ensures an independent, unbiased assessment of the measurement procedure’s performance.^37^

### Conclusion

Laboratory results are crucial in the diagnosis, monitoring and prognostication of patients, and further action often relies on the value of one test result. Laboratories should therefore aim to minimise errors that can affect patient outcomes. The sigma metrics tool has the potential to be a valuable quality management tool for monitoring analytical performance in comparison to world-class standards. However, it is important to set up standardised protocols for the determination of sigma metrics, including choosing the appropriate TEa guidelines and approach to calculating bias.

## References

[1] Jordan B, Mitchell C, Anderson A, Farkas N, Batrla R. The clinical and health economic value of clinical laboratory diagnostics. EJIFCC. 2015;26(1):47–62. 27683481 PMC4975223

[2] Charuruks N. Sigma metrics across the total testing process. Clin Lab Med. 2017;37(1):97–117. 10.1016/j.cll.2016.09.009 28153373

[3] Cooper G. Basic lessons in laboratory quality control. Hercules: Bio-Rad Laboratories; 2008.

[4] Westgard JO. Internal quality control: Planning and implementation strategies. Ann Clin Biochem. 2003;40(6):593–611. 10.1258/000456303770367199 14629798

[5] Shah S, Saini R, Singh SB, Aggarwal O GA. Six Sigma metrics and quality control in clinical laboratory. Int J Med Res Rev. 2014;2(2):140–149. 10.17511/ijmrr.2014.i02.20

[6] Specific criteria for accreditation of medical laboratories (NABL 112; Issue No 04, Issue date 11/02/2019). Haryana: NABL House; 2019.

[7] Litten J. Applying sigma metrics to reduce outliers. Clin Lab Med. 2017;37(1):177–186. 10.1016/j.cll.2016.09.014 28153365

[8] Hens K, Berth M, Armbruster D, Westgard S. Sigma metrics used to assess analytical quality of clinical chemistry assays: Importance of the allowable total error (TEa) target. Clin Chem Lab Med. 2014;52(7):973–980. 10.1515/cclm-2013-1090 24615486

[9] Guo X, Zhang T, Gao X, et al. Sigma metrics for assessing the analytical quality of clinical chemistry assays: A comparison of two approaches. Biochem Medica. 2018;28(2 Special Issue):020708. 10.11613/BM.2018.020708 PMC6039159 30022883

[10] Xia J, Chen S, Xu F, Zhou Y. Quality specifications of routine clinical chemistry methods based on sigma metrics in performance evaluation. J Clin Lab Anal. 2018;32(3):e22284. 10.1002/jcla.22284 PMC6816973 28643351

[11] Ricós C, Garcia-Lario J, Alvarez V, et al. Biological variation database, and quality specifications for imprecision, bias and total error. The 2014 update [homepage on the Internet]. [cited 2020 Jan 08]. Available from: https://www.westgard.com/biodatabase1.htm

[12] U.S Department of Health and Human Services. Medicare, medicaid, and CLIA programs; Regulations implementing the Clinical Laboratory Improvement Amendments of 1988 (CLIA). Federal Register. 1992;57(40):7002–7186. 10170937

[13] Royal College of Pathologists of Australasia. Allowable limits of performance for biochemistry [homepage on the Internet]. [cited 2019 May 05]. Available from: http://www.rcpaqap.com.au/docs/2014/chempath/ALP.pdf

[14] Aarsand AK, Fernandez-Calle P, Webster C, et al. The EFLM biological variation database [homepage on the Internet]. [cited 2020 Apr 30]. Available from: https://biologicalvariation.eu/meta_calculations

[15] Verma M, Dahiya K, Ghalaut V, Dhupper V. Assessment of quality control system by sigma metrics and quality goal index ratio: A roadmap towards preparation for NABL. World J Methodol. 2018;8:44–50. 10.5662/wjm.v8.i3.44 30519539 PMC6275555

[16] Westgard SA. Consolidated comparison of chemistry performance specifications [homepage on the Internet]. [cited 2020 May 13]. Available from: https://www.westgard.com/consolidated-goals-chemistry.htm

[17] El Sharkawy R, Westgard S, Awad AM, et al. Comparison between sigma metrics in four accredited Egyptian medical laboratories in some biochemical tests: An initiative towards sigma calculation harmonization. Biochem Medica. 2018;28(2):233–245. 10.11613/BM.2018.020711 PMC6039160 30022886

[18] Xia Y, Xue H, Yan C, et al. Risk analysis and assessment based on Sigma metrics and intended use. Biochem Medica. 2018;28(2):195–203. 10.11613/BM.2018.020707 PMC6039164 30022882

[19] U.S Department of Health and Human Services. Centers for medicare and medicaid services. Clinical Laboratory Improvement Amendments of 1988 (CLIA) proficiency testing regulations related to analytes and acceptable performance – Proposed changes. Federal Register. 2019;84(23):1564–1566.

[20] Liu Q, Fu M, Yang F, et al. Application of Six Sigma for evaluating the analytical quality of tumor marker assays. J Clin Lab Anal. 2019;33(2):e22682. 10.1002/jcla.22682 30280434 PMC6585744

[21] Aarsand AK, Røraas T, Bartlett WA, et al. Harmonization initiatives in the generation, reporting and application of biological variation data. Clin Chem Lab Med. 2018;56(10):1629–1636. 10.1515/cclm-2018-0058 29596051

[22] Varela B, Pacheco G. Comprehensive evaluation of the internal and external quality control to redefine analytical quality goals. Biochem Medica. 2018;28(2 Special Issue):20710. 10.11613/BM.2018.020710 PMC6039162 30022885

[23] Cao S, Qin X. Application of sigma metrics in assessing the clinical performance of verified versus non-verified reagents for routine biochemical analytes. Biochem Medica. 2018;28(2 Special Issue):20709. 10.11613/BM.2018.020709 PMC6039166 30022884

[24] Li R, Wang T, Gong L, et al. Comparative analysis of calculating sigma metrics by a trueness verification proficiency testing-based approach and an internal quality control data inter-laboratory comparison-based approach. J Clin Lab Anal. 2019;33(9):1–9. 10.1002/jcla.22989 PMC6868403 31386228

[25] Oosterhuis WP, Coskun A. Sigma metrics in laboratory medicine revisited: We are on the right road with the wrong map. Biochem Medica. 2018;28(2):186–194. 10.11613/BM.2018.020503 PMC6039171 30022880

[26] Westgard JO. Useful measures and models for analytical quality management in medical laboratories. Clin Chem Lab Med. 2016;54(2):223–233. 10.1515/cclm-2015-0710 26426893

[27] Tran MTC, Hoang K, Greaves RF. Practical application of biological variation and Sigma metrics quality models to evaluate 20 chemistry analytes on the Beckman Coulter AU680. Clin Biochem. 2016;49(16):1259–1266. 10.1016/j.clinbiochem.2016.08.008 27527571

[28] Westgard JO, Westgard SA. Westgard sigma rules [homepage on the Internet]. [cited 2020 May 13]. Available from: https://www.westgard.com/westgard-sigma-rules.htm

[29] Westgard S, Bayat H, Westgard JO. Analytical Sigma metrics: A review of Six Sigma implementation tools for medical laboratories. Biochem Medica. 2018;28(2):174–185. 10.11613/BM.2018.020502 PMC6039161 30022879

[30] Jones G, Calleja J, Chesher D, et al. Collective opinion paper on a 2013 AACB Workshop of Experts seeking harmonisation of approaches to setting a laboratory quality control policy. Clin Biochem Rev. 2015;36(3):87–95. 26900188 PMC4745611

[31] Van Rossum HH. Moving average quality control: Principles, practical application and future perspectives. Clin Chem Lab Med. 2019;57(6):773–782. 10.1515/cclm-2018-0795 30307894

[32] Gami B, Patel D, Chauhan K, Shah H, Haridas N. Sigma metrics as a quality marker for analyzing electrolytes in the laboratory. Int J Adv Res. 2013;1(7):197–201.

[33] Moya-Salazar J, Pio-Dávila L. Evaluation of inter-batch variability in the establishing and quality control of glucose. Med Univ. 2016;18(71):85–90. 10.1016/j.rmu.2016.03.004

[34] Singh B, Goswami B, Gupta VK, Chawla R, Mallika V. Application of sigma metrics for the assessment of quality assurance in clinical biochemistry laboratory in India: A pilot study. Indian J Clin Biochem. 2011;26(2):131–135. 10.1007/s12291-010-0083-1 22468038 PMC3107403

[35] Zhou B, Wu Y, He H, Li C, Tan L, Cao Y. Practical application of Six Sigma management in analytical biochemistry processes in clinical settings. J Clin Lab Anal. 2020;34(1):1–10. 10.1002/jcla.23126 PMC6977137 31774217

[36] Westgard JO, Westgard SA. The quality of laboratory testing today: An assessment of σ metrics for analytic quality using performance data from proficiency testing surveys and the CLIA criteria for acceptable performance. Am J Clin Pathol. 2006;125(3):343–354. 10.1309/V50H4FRVVWX12C79 16613337

[37] CLSI. Statistical quality control for quantitative measurement procedures: Principles and definitions. 4th ed. CLSI guidelines C24. Wayne, PA: Clinical and Laboratory Standards Institute; 2016.

