# Corrigendum: The application of sigma metrics in the laboratory to assess quality control processes in South Africa

**DOI:** 10.4102/ajlm.v12i1.1996

**Published:** 2023-05-15

**Authors:** Marli van Heerden, Jaya A. George, Siyabonga Khoza

**Affiliations:** 1 National Health Laboratory Service, Johannesburg, South Africa; 2 Faculty of Health Sciences, Charlotte Maxeke Johannesburg Academic Hospital, University of the Witwatersrand, Johannesburg, South Africa

In the published article, Van Heerden M, George JA, Khoza S. The application of sigma metrics in the laboratory to assess quality control processes in South Africa. Afr J Lab Med. 2022;11(1):a1344. https://doi.org/10.4102/ajlm.v11i1.1344, there was an error. The incorrect Sigma metric calculation was stated.

The correction has now been made on page 2 in the Methods section, under Data collection and analysis, paragraph 4 and should read:

**The original paragraph:**

Sigma metrics were calculated for each analyte on two levels as follows:

Sigma=TEa−(bias/standard deviation). [Eqn 3]

**The revised and updated paragraph:**

Sigma metrics were calculated for each analyte on two levels as follows:

Sigma=(TEa−bias)/standard deviation. [Eqn 3]

The authors apologise for this error. The correction does not change the study’s findings of significance or overall interpretation of the study’s results or the scientific conclusions of the article in any way.

